# An Overview of B-1 Cells as Antigen-Presenting Cells

**DOI:** 10.3389/fimmu.2016.00138

**Published:** 2016-04-11

**Authors:** Ana F. Popi, Ieda M. Longo-Maugéri, Mario Mariano

**Affiliations:** ^1^Departamento de Microbiologia, Imunologia e Parasitologia, Escola Paulista de Medicina, Universidade Federal de São Paulo, São Paulo, Brazil

**Keywords:** B-1 cells, APCs, BCR, TLR, autoimmune diseases

## Abstract

The role of B cells as antigen-presenting cells (APCs) has been extensively studied, mainly in relation to the activation of memory T cells. Considering the B cell subtypes, the role of B-1 cells as APCs is beginning to be explored. Initially, it was described that B-1 cells are activated preferentially by T-independent antigens. However, some reports demonstrated that these cells are also involved in a T-dependent response. The aim of this review is to summarize information about the ability of B-1 cells to play a role as APCs and to briefly discuss the role of the BCR and toll-like receptor signals in this process. Furthermore, some characteristics of B-1 cells, such as natural IgM production and phagocytic ability, could interfere in the participation of these cells in the onset of an adaptive response.

## Introduction

Murine B-1 cells are known as innate B lymphocytes. However, the classification of these cells as B cells raised many questions, including whether this B cell subtype works in a similar or different way than its counterpart, conventional B-2 cells. Several reviews discuss differences between these two cell populations, regarding to their ontogeny, anatomical localization, antibody repertoire, antigen stimulus, and role in the immune response (1–6). The two major subsets of B cells, B-2 and B-1 cells, are defined by the differential expression of CD5 (7). Along with the presence of CD5 on their surface, B-1 cells are further differentiated from B-2 cells by the expression of CD11b, IgM^high^, IgD^low^, and the absence of CD23 (8). Furthermore, CD5 expression also subdivides the B-1 cells into two different subsets, B-1a cells, which are CD5^+^, and B-1b cells, which are CD5^−^ (2, 8). B-2 cells are produced in the bone marrow from hematopoietic stem cells and migrate to the secondary lymphoid organs as immature B cells. In these organs, they differentiate into follicular and marginal zone B cells (9, 10). B-1 cells are mainly present in the peritoneal and pleural cavities and constitute only a small fraction of the B cells in the spleen (11). B-1 cell origin and development occur primarily during the fetal stage from distinct precursors from B-2 cells (12, 13). Recently, Ghosn et al. (14) described that HSCs sorted from adult bone marrow and transferred to lethally irradiated recipients clearly give rise to B-2 and B-1b cells but do not detectably reconstitute B-1a cells (14). Furthermore, it has been postulated that the B-1a lineage derives independently from a hematopoietic stem cell (14). These B cell subsets specialize in the recognition of diverse antigens; consequently, they provide distinct immune effector functions. B-1a cells play a role in innate immunity *via* their contribution to natural antibodies, whereas B-1b cells are critical in the development of IgM memory cells (1). B-1a cells respond rapidly to T-cell-independent antigen (15). B-1a cells are also known to produce most of the natural antibodies in the serum (16, 17). Despite this, B-1 cell antibodies have been found to be reactive to self-antigens, and hyperplasia of the B-1 cell population has been found in some autoimmune diseases (18, 19). The antibody production by B-1b cells has been poorly investigated. By contrast, the B-2 cell response to protein antigens is well described and elicits a T-cell-dependent immune response.

There are few reports about the possible roles each B cell subtype exerts in the immune response by acting as APCs. Although the majority of articles indicate the participation of B-1 cells in recognizing the T-cell-independent antigen, some reports demonstrate their role as antigen-presenting cells (APCs) (20–25). This role is extremely important because it could be one of the functions that have allowed the maintenance of B-1 cells through phylogenetic evolution. Furthermore, a more comprehensive status regarding this function could provide explanations concerning the role of B-1 cells in the immune response and in some diseases, such as autoimmune diseases.

## Antigen-Presenting B-1 Cells

Ron et al. (26) first demonstrated evidence of the role of B-2 cells in the CD4^+^ T cell response by showing a failure of proliferative T cell responses to protein antigens in B cell-depleted mice. To determine whether B cell deficiency caused the T cell response impairment in these mice, the authors showed that splenic cells and peritoneal macrophages were able to stimulate T cell response *in vitro*. Although the first description of this occurred in the beginning of the 80s, the priming of T cells by B lymphocytes remains controversial (26–30), and scarce information exists about the role of other B cell subtypes. It has been postulated that the antigen-presentation capacity of antigen-specific B-2 cells is reserved for the re-activation of memory T cells (29, 31, 32). This could be, in part, attributed to the kinetics of the response of B-2 cells, considering that the activation of these cells by a specific antigen takes several days. In this case, activated B-2 cells might encounter their cognate primed T cell and then play a role in their function as APCs (33–35). In this condition, activated B-2 cells are optimal APCs because they drive the T lymphocyte response to specific antigens (36, 37) and are able to modulate the polarization of T cells by cytokines.

As mentioned before, B-1 cells respond to T-independent antigens and are responsible for the production of natural antibodies, mostly IgM. These cells do not effectively participate in T-dependent responses [reviewed by Berland and Wortis (1)]. Despite this, some reports provide evidence that B-1 cells also stimulate T cells and respond to T-dependent antigens (20). Combined adoptive transfer experiments of OVA-pulsed peritoneal-derived B-1 cells and CFSE-labeled T cells demonstrated that B-1 cells are able to stimulate T cell proliferation. Furthermore, antigen presentation by B-1 cells to OVA-specific T cells was also demonstrated *in vitro* (20). Constitutive expression of MHC class-II, CD80, and CD86 by B-1 cells validated these findings (22). Furthermore, the presence of an inflammatory stimulus or a specific antigen augments these molecules on the surface of B-1 cells (22, 38, 39). Zimecki and Kapp (24) and Zimecki et al. (25) showed that B-1 cells present Ags to Ag-specific T cells and induced more efficient proliferation than conventional B cells.

## BCR and TLR as Antigen Uptake Players on B-1 Cells

B cells have two primary pathways for their activation as APCs, which occurs through BCR or the germline-encoded PAMP receptors (40–42). BCR plays a dual role in B-2 cell activation: (1) the ligation of specific antigens in the BCR induces a signaling cascade that leads to the activation and proliferation of B-2 cells (43) and (2) the BCR–antigen interaction results in internalization and processing of the antigen. Although they are not completely elucidated, the BCR signals in B-1 cells are quite different than in B-2 cells (44–46). B-1 cells show a failure to be activated after BCR engagement, and multiple mechanisms appear to be involved in maintaining B-1 cells in an anergic state. One such mechanism involves Lyn, which acts by phosphorylating ITIMs on inhibitory receptors, leading to the recruitment of PTPs that antagonize the BCR-mediated activation of PTKs. IL-10 also plays a key role in controlling the expansion of self-reactive B-1 cells. CD5 was also indicated as a negative regulator of BCR signals in B-1 cells. Defects in the negative regulatory mechanisms may account for the accumulation of B-1 cells and autoantibodies in autoimmune diseases. However, in an infectious disease, signals from CD40 and high-dose TLR ligands can overcome the anergic state of B-1 cells, enabling their activation during infection (44–46).

Interestingly, in addition to the fact that a non-functional BCR results in a defect in the activation of B-2 cells, it also causes a failure in the T cell response (26). This information supports the idea that internalization of the antigen by the BCR is important to the APC function of B-2 cells. It has been demonstrated that the absence of B cell antigen presentation, due to the lack of MHC expression or a non-functional BCR, results in a defect in the memory CD4 response. Barr et al. (40) demonstrated that the TLR activation of B-2 cells is important for the generation of the primary Th1 response in an antigen presentation-independent process. However, BCR recognition and B cell antigen presentation are absolutely required for the development of Th1 memory cells and hence confer protective immunity to *Salmonella*. With respect to B-1 cells, Gao et al. (47) demonstrated that the antigen specificity of the BCR was involved in the uptake of *Salmonella* by B-1 cells, and the number of phagocytic peritoneal B-1 cells from TgVH3B4 mice was almost threefold higher than that observed in the littermate control mice. Based on these results, it could be suggested that the BCR is essential for the phagocytosis of bacteria by B-1 cells and is also important for enabling these cells to exert APC functions.

Conversely, specific antigen uptake by the BCR and the activation of B cells by the TLRs connect them to an innate phase of the immune response. B-2 cells express many of the TLRs (42, 48, 49), and TLR4 and TLR9 are the most studied. TLR expression is quite different among the B cells subsets. B-2 cells express less TLR2, 6, and 7 and equal amounts of TLR1, 4, 5, and 9 compared to other B cell subsets. Interestingly, MZ B cells and B-1 cells show a clear similarity in TLR expression, displaying increased levels of TLR2, 6, and 7, along with decreased levels of TLR8 (50).

Intriguingly, differences regarding their response to TLR agonists were found between naive murine B cell subsets. Triggering of the TLR induces B-1, but not B-2, cells to differentiate into fully mature plasma cells (51). Based on these data, the authors proposed that during a natural infection, the TLR stimulation leads to the production of protective natural antibodies by the B-1 cells, which could control the microbial load until the adaptive arm of the immune response becomes fully functional.

It is well known that LPS functions as a B-2 cell mitogen, leading to the activation and polyclonal expansion of these cells, independent of BCR reactivity. *In vitro*, B-1 cells proliferate and produce antibodies in response to TLR engagement. Certain TLR ligands, including Pam3CSK (TLR1/TLR2), MALP2 (TLR2/TLR6), LPS (TLR4), and R848 (TLR7/TLR8), are able to induce B-1 cell proliferation, whereas others, including flagellin (TLR5) and polyIC (TLR3), are not (51). The proliferation of B-1 cells in response to LPS is stronger than in B-2 cells; however, it is less intense in response to CpG.

Some reports show evidence that stimulation of the TLRs in B cells impacts in the T cell response *via* not only the secretion of diverse cytokines but also the increased expression of several costimulatory molecules involved in the T:B cell interaction, such as MHC-II, CD40, CD80, CD86, and others (42, 49, 52–54). Interestingly, our group has described the adjuvant effect of *Propionibacterium acnes* on the expression of the TLR by B-1 cells (39) (Gambero et al., submitted). *P. acnes*, a Gram-positive bacillus, is the major constituent of the normal human adult skin microflora (55). As already demonstrated in clinical and experimental models, a dead *P. acnes* suspension modulates the innate and acquired immune responses, including an increase in antibody responses and phagocytic and tumoricidal macrophage functions (56–63). An important effect of *P. acnes* is its capacity to direct the immune response toward a Th1 or Th2 response (64, 65). *P. acnes* interferes in the B-1 cell APC activity through the toll-like receptors (TLRs), principally TLR2 and TLR9 (66–68). Interestingly, the bacterium increased the expression of MHC-II, CD80, CD86, CD40, TLR2, TLR4, and TLR9 by the B-1 cells (39). It was also confirmed that the adjuvant effect induced by *P. acnes* on the B-1 cells is mediated by TLR2 (Gambero et al., submitted). *P. acnes* increases the number of B-1 cells *in vitro* and *in vivo*, induces their early differentiation into phagocytes *in vitro*, and increases the phagocytic ability of these cells (39) (Gambero et al., submitted). The direct influence of *P. acnes* on antigen presentation by B-1 cells is under investigation. Furthermore, LPS stimulus also increases the differentiation of B-1 cells into phagocytes *in vitro* and *in vivo* (69, 70); however, whether the stimulus increases the APC activity is not yet clear.

Another important point in TLR signaling in B cells is cytokine production. Barr et al. (41) demonstrated that the binding of TLR2, TLR4, and TLR9 in B cells induces B-2 cells to secrete IFN-gamma and IL-6. Interestingly, TLR engagement in B-1 cells augmented the secretion of the proinflammatory cytokine IL-6 and the anti-inflammatory cytokine IL-10 (71), which have antagonistic effects in the course of the immune response. However, both cytokines are important to B-1 cell proliferation and viability. IL-6 is related to antibody production by B-1 cells in autoimmune disease, and IL-10 plays a negative role in the activation of the BCR. It could be speculated that the secretion of both cytokines after TLR signals could be important in the control of the B-1 cell response in the onset of inflammation and could prevent the development of an exacerbated response to self-antigens.

## The Role of CD80/CD86 in B-1 Cells

Some studies point to the role of certain molecules in the induction of CD4^+^ T cell polarization by B-1 cells. B-1 cells express elevated levels of CD80 and CD86 in comparison to B-2 cells (72). Zhong et al. (73) demonstrated that CD86 blockage markedly reduced the capacity of B-1 cells to stimulate alloreactive T cells. Interestingly, the same treatment increased the generation of Treg cells after antigen presentation by B-1 cells. However, anti-CD80 antibody treatment had much less of an effect on the capacity of B-1 cells to stimulate alloreactive T cells and on the generation of Treg cells than did anti-CD86 antibody treatment (73). Anti-CD86 treatment also partially inhibited the induction of Th17 cells (23). In agreement with these findings, De Lorenzo et al. (38) demonstrated that OVA-presensitized B-1 cells were less able to induce T cell proliferation than naive B-1 cells. Additionally, these authors described that OVA-presensitized B-1 cells express high levels of MHC-II molecules compared to naive B-1 cells and that the expression of CD86 was lower.

Interestingly, the CD86 molecule may also provide the costimulatory signal in fish B cell, similar to B-1 cells (74). These results indicate that even in primitive vertebrates, B cells may act as pivotal initiating APCs in priming naive T cells, similar to DCs and macrophages in mammals. Elucidating how antigen presentation occurs in ancient vertebrates and in different B cell subtypes in mammals may contribute to the understanding of the evolutionary history of B cell populations, such as the origin of the B-1 subset, and of the evolution of the adaptive immune response.

## T Cell Polarization by B-1 Cells

Although some authors have described the production of cytokines by B-1 cells, scarce literature exists about the polarization of T cells by them. Some reports about this pointed out that B-1 cells favor Th17 polarization (23, 73).

Comparing the APC activity of B-2 cells and peritoneal B-1 cells, Margry et al. (20) demonstrated that peritoneal B-1a cells increase the percentage of IL-10-, IFN-γ-, and IL-4-producing T cells. Furthermore, peritoneal B-1a cells present antigens to CD4^+^ T cells in the peritoneal cavity, which is totally distinct from the milieu, where antigen presentation by conventional B cells occurs. The authors also show that the activation of CD4^+^ T cells by peritoneal B-1a cells promotes a more intense proliferation compared to conventional B cells. Although peritoneal B-1a cells induce activated T cells to produce larger amounts of IL-10, IFN-γ, and IL-4 *in vitro* (20), B-1a cells derived from the spleen preferentially induce IL-17 production by T cells. In agreement with this, other authors also demonstrated that B-1 cells preferentially induce Th1 and Th17 differentiation *in vivo* (73). This strong inflammatory response elicited by the B-1 cells could be related to their role in the autoimmune diseases (21). Interestingly, in cultures for the generation of Treg cells, when B-1 cells were used as the APCs, fewer Foxp3^+^ Treg cells were generated in comparison to cultures where splenic B-2 cells were used (73). The failure to generate Treg cells, in addition to the induction of a Th1/Th17 profile by the B-1 cells, could be one of the mechanisms of the autoimmune profile of B-1 cells. However, this hypothesis is only a speculation, because the induction of the Th profile by the B-1 cells needs to be investigated in more detail and in different infection/disease models.

Other important point to be discussed is about the localization of antigen presentation by B-1 cells. Despite the migration of B-1 cells to inflammatory milieu and other organs (75–77), it was not well documented if it is necessary that these cells migrate to secondary lymphoid organs to exert APC role. Interestingly, Margry et al. (20) demonstrated that peripheral T cells visit the peritoneal cavity and engage with antigen presented by B-1 cells in this location. In this context, we could postulate that APC role of B-1 cells could be more direct to reactivate memory T cells than priming naive T cell since that the majority of T cells found in the peritoneal cavity is memory T cells.

## The Role of B-1 Cell Antigen Presentation in Autoimmune Diseases

As mentioned before and in the literature, the TLR and BCR signals are important in the assembly of the adaptive response. In an early stage, TLR signals drive the secretion of cytokines by the APCs and contribute to the expression of some molecules that are important in the antigen-presenting process. Later, BCR-mediated uptake is important to the antigen presentation by B cells and the maintenance of memory T cells. In addition to its importance in the secondary response during an infection, this also could be a mechanism that induces autoimmune disease. In fact, the involvement of BCR/TLR signals in driving T cells in lupus (78) and EAE has already been described (21, 79).

Sato et al. (21) described an increase in the B-1 cell population in the disease target organs of a murine model of lupus. These authors demonstrated that B-1 cells aberrantly migrate into the thymus during the development of lupus nephritis and that B-1, but not B-2, cells induce the activation and expansion of thymic CD4^+^ T cells in the presence of IL-2. Considering that B-1 cells often recognize self-antigens and express higher levels of costimulatory molecules, their role in activating the autoreactive TCR should be considered. These findings may provide a novel understanding of the mechanism for the loss of immunological tolerance in the development of autoimmune disease.

## Concluding Remarks

B-1 cells have peculiar features, such as a mixture of innate and lymphoid cell properties. As mentioned here, some authors describe similarities between B-1 cells and primordial B cells in ancient vertebrates (74). As described for teleost fish B cells, B-1 cells are phagocytic cells (6, 69, 70, 75) and have microbicidal activity (80, 81), similar to DCs and macrophages in mammals. Furthermore, several reports indicate that B-1 cells may play a role as initiating APCs, not only in the maintenance of memory T cells, similar to conventional B cells, but also in priming CD4^+^ T cells. It is important to reinforce that B-1 cells produce a majority of the IgM natural antibodies. In this context, we postulate that these antibodies could form immunocomplexes with self-antigens and that B-1 cells could internalize these, leading to intense antigen presentation to T cells. A speculative scenario was proposed, in a continuous inflammatory stimulus, elevated levels of IL-6 increased induce expansion of B-1 cell population, and also increased the B-1-cell antibody production. Furthermore, the CD86 expression by B-1 cells is also augmented, and it favors the B-1 cell APCs function and also the induction of Th17 profile. Considering that B-1 cells produce mainly self-reactive antibodies, the increased levels of the B-1 cell-derived antibodies could leave to an increase in the antigen uptake by these cells and also augment in antigen presentation to self-reactive TCR, which could lead finally to an induction of autoimmune diseases.

In conclusion, further detailed investigations should be conducted to elucidate the role of B-1 cells in priming T cells during an infectious disease. Several strategies have been developed considering the use of APCs in therapies for which amplification of the immune response is necessary; thus, B-1 cells might also be considered in this context. Furthermore, the participation of these cells in the breakdown of self-tolerance should also be considered.

## Author Contributions

AFP conceived the idea and topic of the review, wrote and discussed the text with other authors. IMLM and MM discussed the ideas, read and made suggestions in the final text.

## Conflict of Interest Statement

The authors declare that the research was conducted in the absence of any commercial or financial relationships that could be construed as a potential conflict of interest.
